# The Wear of Seal Fins during High-Speed Rub between Labyrinth Seal Fins and Honeycomb Stators at Different Incursion Rates

**DOI:** 10.3390/ma14040979

**Published:** 2021-02-19

**Authors:** Bin Lu, Xiaojian Ma, Caiguang Wu, Haijun Xuan, Weirong Hong

**Affiliations:** 1 High-Speed Rotating Machinery Laboratory, College of Energy Engineering, Zhejiang University, Hangzhou 310027, China; 11628018@zju.edu.cn (B.L.); hongwr@zju.edu.cn (W.H.); 2 Shenyang Engine Research Institute, Aero Engine Corporation of China, Shenyang 110015, China; 13842016492@163.com (X.M.); wcg7321@sina.com (C.W.)

**Keywords:** labyrinth–honeycomb seal, rubbing behavior, Ti17, wear mechanism

## Abstract

Labyrinth seals as a noncontact sealing technology are widely used in aero-engine. To improve the efficiency of the aero-engine, the clearance between the rotor and stator must be as small as possible. However, the change of the clearance between the rotor and stator because of thermal expansion, vibration, mechanical loading may lead to undesirable high-speed rub, which will lead to the cracking of the seal fins. This paper focuses on the wear of the seal fin after the rub and presents the rubbing tests between seal fins and the metal honeycomb under rubbing speed of 380 m/s and incursion rates between 20 and 180 μm/s, with an incursion depth of 1500 μm and a temperature of 350 °C. The rubbing force and temperature were recorded, and the seal fins were checked by SEM and EDS. The results show that the wear mechanism of seal fins changed from oxidation wear and adhesive wear to delamination wear and then to metal wear with the increasing incursion rate. The axial cracks appeared on the worn surface of the seal fins due to the cracking of tribo-layers under periodic thermomechanical stress. The wear mechanism of the seal fin also has a great influence on the rubbing force and temperature.

## 1. Introduction

Increasing engine effectiveness and reducing leakages are the main goals for the development of aero-engines. Various sealing technologies have been developed and applied to aero-engine to reduce leakages. Labyrinth seals, which have several teeth on the rotation part, as noncontact sealing technology are still widely used in aero-engines [1,2]. They have many advantages, such as a simple design, long lifetime, and applicability under extreme operating conditions [3]. Metal honeycomb is commonly used in labyrinth seals as the abradable stator, mainly because the contact area of the honeycomb is small. Choosing the appropriate materials, such as Hastelloy X, can result in the honeycomb having good erosion, corrosion, and oxidation resistances. These characteristics are important to maintain the mechanical properties of the honeycomb under extreme operating conditions [4]. A significant method of improving the engine efficiency and lessening fuel consumption is reducing the clearance between the rotor and stator [5]. However, during the operating cycle of the aero-engine, the change of the clearance between the rotor and stator because of thermal expansion, vibration, mechanical loading may lead to undesirable high-speed rub [6]. Excessive heat and forces generated during the undesirable rub may lead to the cracking of labyrinth seal fins, which will threaten the security of the aero-engine [7].

Investigating the high-speed rubbing behaviors between the labyrinth seal fins and honeycomb stators is significant to choose the appropriate abradable materials and optimize the labyrinth seal design. The ultimate goal is to reduce the seal fin wear and the risk of crack generation on the seal fin to ensure safety of the aero-engine. The complexity of the high-speed rub leading to experimental study is still the main research method. Today, relatively little experimental data are available about the rub behaviors between labyrinth seal fins and honeycomb stators. Bill and Shiembob [8] compared the performances of Hastelloy X honeycomb with and without an aluminide coating. The results demonstrated that aluminide-coated honeycomb tended to stick more to the seal fins during the rub, and the wear of the seal fin was uneven. Sporer et al. [9] executed rubbing tests using a knife edge ring and a test blade to make contact with honeycomb. They mainly researched the abradability of honeycombs made of different materials by measuring knife edge wear. Rathmann et al. [10] presented rubbing tests to research the rubbing behaviors between labyrinth seal fins and honeycomb. However, no detailed data were presented. High-speed rubbing tests using a dummy blade with two seal fins to make contact with honeycomb were carried out by Zhang et al. [11]. They found that maximum rubbing forces were not proportional to the incursion rate, and smearing appeared on the worn surface of honeycomb. Pychynski et al. [12,13] idealized the contact of a honeycomb seal with the contact between a single metal foil and a seal fin to reduce the complexity of the research. The contact forces, friction temperature, and wear were measured. The results showed that the rubbing forces decreased with the rubbing speed, and the rubbing temperature was around 800 °C. Zhang et al. [14] investigated the rubbing behaviors of the honeycomb with a nickel–aluminide filler in a labyrinth seal system. They pointed out that the nickel–aluminide filler with high aluminium content made the honeycomb hard to fracture, but easy to fracture after ageing. The fin was less worn when the honeycomb was easy to fracture.

All the research mentioned above mainly focused on the rubbing behaviors at different rubbing conditions and evaluated the abradability of the honeycomb. However, the wear of the seal fin has not been detailed studied. In the aero-engine, labyrinth seal fins are in an environment of high speed and high temperature. The wear of the seal fin may lead to crack generation and propagation on the rotor, which will threaten the safety of the aero-engine. Hence, the wear of the seal fin after the rub should be paid more attention. The object of this paper was the wear of the seal fin caused by the high-speed rub between the labyrinth seal fin and the honeycomb in high pressure compressor interstage seals. The rub test conditions (rub speed and temperature) presented in this paper are similar to those in high pressure compressor interstage seals, so that the test results can better reflect the real situation. The aim of this paper was to improve the understanding of the wear of seal fins at different incursion rates. 

## 2. Materials and Methods

### 2.1. Test Rig

**Figure 1:**
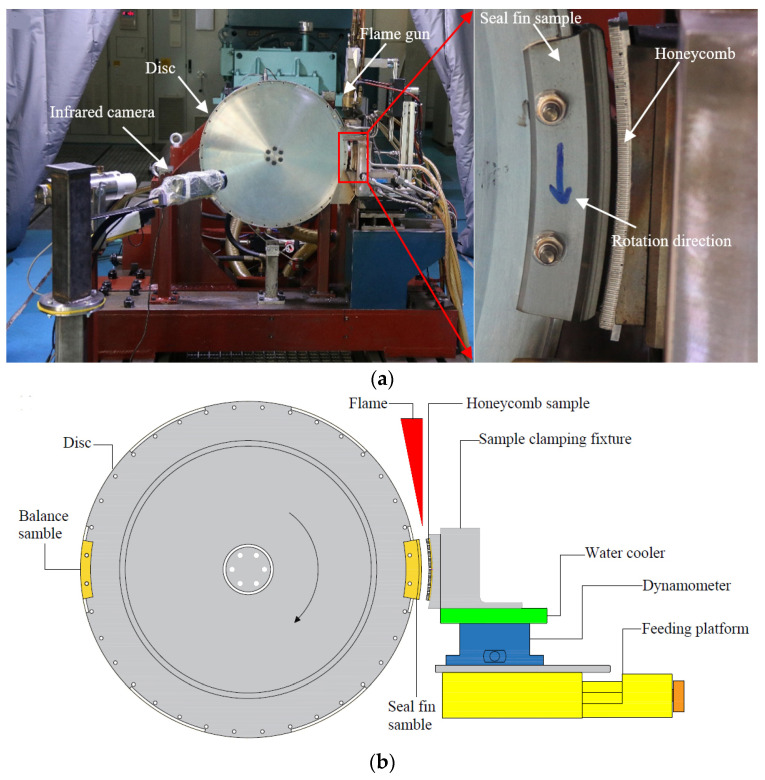
(**a**) Test rig; (**b**) test rig schematic diagram.

The test rig used to run the rub tests, which had a maximum rotation speed of 15,000 rpm, is shown in Figure 1a. The test rig schematic diagram is shown in Figure 1b. The feeding platform enabled the radial incursion of the honeycomb sample into the seal fins at a defined incursion rate up to a pre-set final incursion depth. A flame gun was used to heat the honeycomb to simulate the high temperature environment in an aero-engine. The flame gun was mounted on the feeding platform so that the flame gun and the honeycomb could move simultaneously and the relative position of the flame gun and the honeycomb remained constant during the rub. The fuels of the flame were propane and oxygen. Before the rub test, a temperature calibration test was performed to determine the flow rate of oxygen and propane needed to heat the honeycomb to 350 °C. A dynamometer (Type 9257B, Kistler, Winterthur, Switzerland) was positioned underneath the honeycomb sample to measure the rubbing force. The force data were recorded by a high-speed acquisition device with an acquisition frequency of 100 kHz. An infrared camera FILR A615 (Wilsonville, OR, USA) was used to measure the instantaneous rubbing temperature on the honeycomb with an acquisition frequency of 100 Hz.

### 2.2. Test Samples

**Figure 2:**
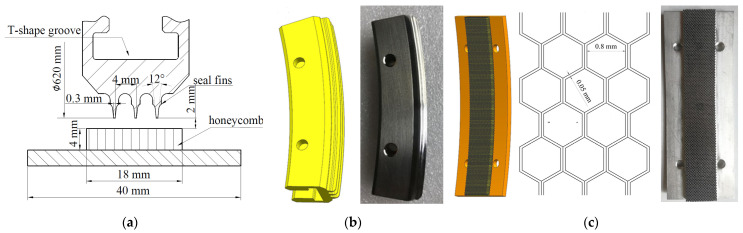
(**a**) The geometries of seal fins; (**b**) the seal fin sample and; (**c**) the honeycomb sample and honeycomb structure.

In high pressure compressors, there are seal fins on the circumference of the rotor. However, a seal fin ring would greatly increase the test cost. In order to facilitate the seal fin check after the rub and reduce the test cost, a special adapter disc and arc seal fin sample were designed. Three seal fins with the same geometry had been manufactured onto the seal fin sample, shown in Figure 2a. For the convenience of checking the seal fin, the seal fin sample was processed into an arc block. The rub between the seal fin and honeycomb is more like grinding than cutting. If the angle of the seal fin block is too small, the rub is more like cutting, while if the angle of the seal fin block is too big, the test cost will increase greatly. As a result, the angle of the seal fin sample was set to 20°, as shown in Figure 2b. A seal fin ring was first manufactured because the T-shape groove and the seal fins were hard to process, and then the seal fin ring was cut into 18 seal fin samples. The rubbing speed is an important parameter that has great influence on the rubbing behaviors. As a result, the rubbing speed presented here was set to 380 m/s, which is consistent with the rubbing speed of the seal fin in high pressure compressor. Considering the performance and the safety of the test rig, the diameters of the seal fins were set to 620 mm, corresponding to a rotor speed of 11,711 rpm. The seal fin sample was assembled to the disc by a T-shape groove and two bolts shown in Figure 1a. The same arc block without fins was mounted opposite the seal fin sample for balance. The honeycomb sample used in the tests and a diagram of the honeycomb structure are shown in Figure 2c. The honeycomb was made from 0.05 mm-thin sheets which were spot-welded together to form the periodic hexagonal cell structure. The regular hexagonal cell size was 0.8 mm. The diameter of honeycomb surface was 624 mm. The honeycomb sample was also arcuate, with an angle of 20°.

The tests presented in this paper simulated the rub in a high-pressure compressor. The materials chosen in the test were the same as the material in an aero-engine. Therefore, the selected material combinations were Ti17 (Ti-5AI-2Sn-2Zr-4Mo-4Cr) for seal fins and Hastelloy X for honeycomb sample. Hastelloy X is a typical honeycomb material, and Ti17 is commonly used in high pressure compressor for its low density and high strength. The compositions of these materials are shown in Table 1 and Table 2.

### 2.3. Test Procedure

**Figure 3:**
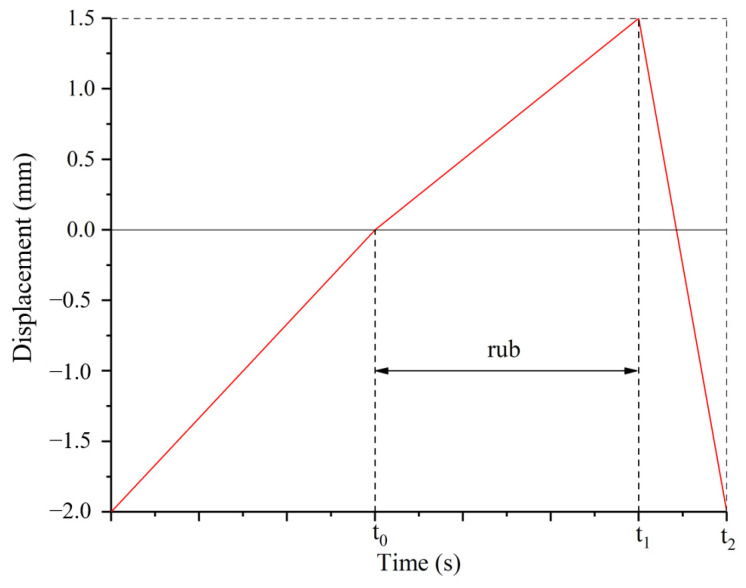
Typical incursion profile.

Before the test, the distance between the seal fin sample and the honeycomb sample was about 2 mm. First, turned on the electric motor to drive the rotor. During the acceleration, ignited the flame gun to heat the honeycomb. The feeding platform pushed the honeycomb forward in radial direction at a speed of 50 μm/s when the rotor speed and temperature were stable. When the seal fins had contact with the honeycomb, the platform would move to the preset incursion depth at preset incursion rate. Once the final incursion depth was reached, the feeding platform retracted quickly to prevent further contact. A typical incursion profile for a rub test is shown in Figure 3. During the rub, rubbing force and temperature were recorded simultaneously. The seal fin sample and the honeycomb sample were replaced after one rub test. The seal fins after the rub tests were checked by Scanning Electron Microscope (SEM, Zeiss, Oberkochen, Germany) and Energy Dispersive Spectrometer (EDS, Zeiss, Oberkochen, Germany) to analyze the wear mechanism and damage. The mass change of the seal fin was measured by an electronic balance with an accuracy of 1 mg before and after the rub test to calculate the wear rate of the seal fin.

### 2.4. Test Parameters

In the tests, the rubbing speed, temperature, and incursion depth were kept constant. The research focused on the effect of incursion rate on the wear of the seal fin. The test matrix is shown in Table 3. The temperature and rubbing speed were the same as the rub in the high-pressure compressor. The depth of the honeycomb wear groove in the aero-engine is close to 1500 μm. As a result, the incursion depth in the test was set as 1500 μm. The incursion rate measured in aero-engine is close to 100 μm/s from engine start to normal operation. Hence, the incursion rates in the tests were set near 100 μm/s, and the interval of incursion rate was 40 μm/s.

## 3. Results

To analyze the wear of the seal fin, the rubbing force and temperature, wear rate worn morphology, cross-sectional morphology and EDS results are presented in this section.

### 3.1. Rubbing Force and Temperature

**Figure 4:**
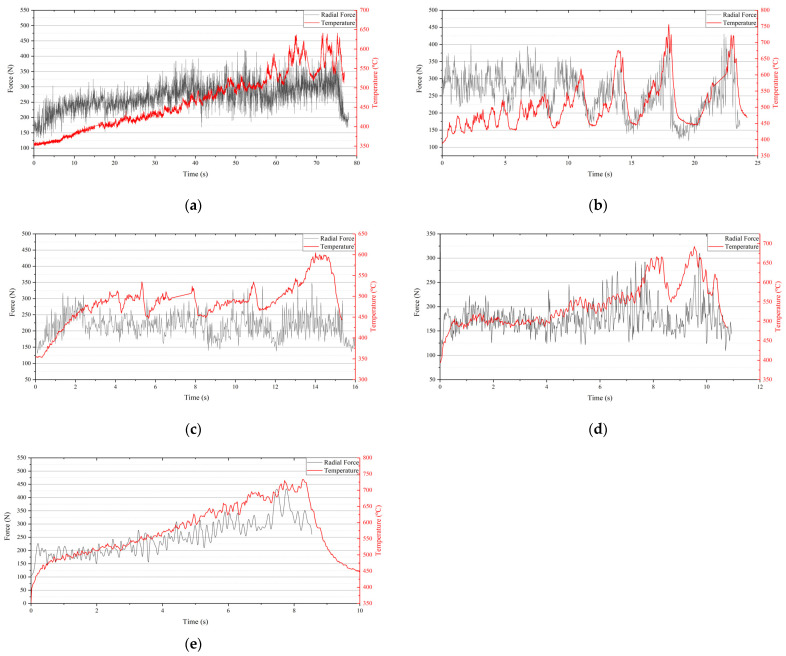
Rubbing forces and temperature: (**a**) 20 µm/s; (**b**) 60 µm/s; (**c**) 100 µm/s; (**d**) 140 µm/s; (**e**) 180 µm/s.

The radial rubbing force and the rubbing temperature at different incursion rates are shown in Figure 4. The radial force curves shown in Figure 4 are envelopes extracted from the original data to make the image more concise. On the whole, the radial force and the temperature increased with the rubbing time. The profiles of the rubbing force and the temperature at different incursion rates had different characteristics. At 20 µm/s, the radial force and temperature increased with small peaks with the rubbing time before 60s. After that, large fluctuations appeared in the curves. At 60 µm/s, the fluctuation was obvious at the beginning of the rub. Large peaks occurred periodically. At 100 µm/s, there were no obvious peaks at the beginning of the rub. As the rubbing time increased, the large peaks appeared and the durations of the troughs were shorter than those at 60 µm/s. At 140 µm/s, the curves were flat and only small peaks appeared on the curves when the rubbing time was less than 8 s. Two large peaks appeared at the end of the rub. At 180 µm/s, no large fluctuation appeared on the curves. The radial force and temperature continuously increased as the rubbing time increased.

### 3.2. Wear Rate

**Figure 5:**
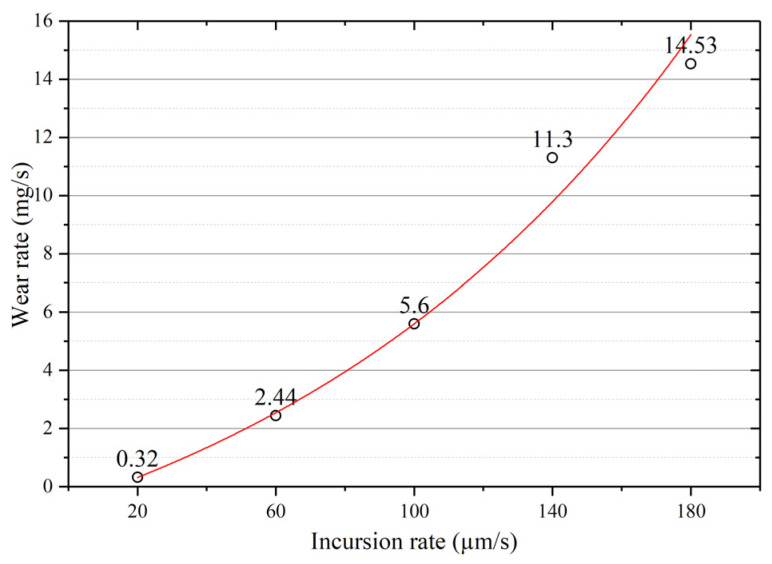
Wear rate of the seal fins as a function of the incursion rate.

The incursion depth was constant, and the rubbing time was different at different incursion rates; therefore, the wear rate here was defined as the wear loss of the seal fin per second. The wear rate of the seal fin as a function of the incursion rate is shown in Figure 5. At the incursion rate of 20 µm/s, the wear rate was small, which indicates that there was mild wear on the seal fins. With the increasing incursion rate, the wear rate continuously increased, which means that the wear of the seal fin progressed from mild wear to severe wear. The wear rate showed an exponential growth trend.

### 3.3. Worn Morphology

**Figure 6:**
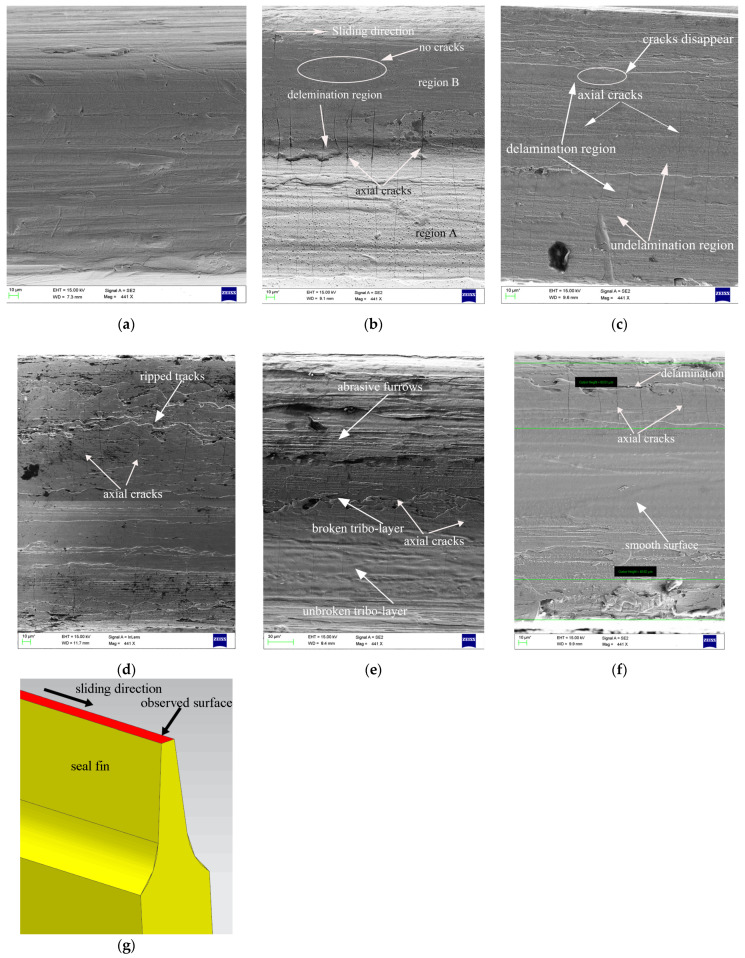
Worn surfaces of the seal fins at different incursion rates: (**a**) the original seal fin; (**b**) 20 µm/s; (**c**) 60 µm/s; (**d**) 100 µm/s; (**e**) 140 µm/s; (**f**) 180 µm/s; (**g**) observed surface.

The worn surfaces of the top of the seal fins were observed by SEM (red surface in Figure 6g). The worn surfaces at different incursion rates present different features. At 20 μm/s, as shown in Figure 6b, region A is relatively white while region B is dark, which indicates that region A and region B are not in the same plane. The surface between region A and region B shows broken features, which indicates delamination of the tribo-layer. However, the delamination of the tribo-layer is limited, and most of the worn surface is smooth and intact. At 60 μm/s, the worn surface is relatively flat. The delamination wear is obvious, causing the continuous delamination region on the worn surface shown in Figure 6c. The surface of the delamination region is very smooth without any furrows, which indicates that this region lost contact at a time during the rub because the delamination region is lower than the undelamination region. At 100 μm/s, shown in Figure 6d, the edge of the worn surface presents the rapped tracks due to the delamination of the tribo-layer. The middle region of the worn surface is smooth, attributed to the rapid wear of the tribo-layer. At 140 μm/s, shown in Figure 6e, the worn surface is very rough, with a broken tribo-layer and abrasive furrows. The formation of the abrasive furrows can be attributed to the hard particles from the broken tribo-layer. At 180 μm/s, shown in Figure 6f, the middle region of the worn surface is very smooth with some melting features. At the edge of the worn surface, delamination of the tribo-layer also occurs. 

Another typical feature is that the cracks perpendicular to the sliding direction appear on the worn surface. These cracks are called axial cracks in this paper. At 20 μm/s, the axial cracks are obvious at region A. Most of the axial cracks do not pass through region B, as shown in Figure 6b. At 60 μm/s, the axial cracks run through the entire surface. The axial cracks are more obvious at the undelamination region. Some of the axial cracks disappear at the delamination region. At 100 μm/s, the shallow and narrow axial cracks only appear on the edge of the worn surface, where the tribo-layer is severely delaminated. At the middle region of the worn surface, where the surface is smooth, no axial cracks appear. At 140 μm/s, the axial cracks again run through the entire surface, although the delamination wear of the tribo-layer is severe. At 180 μm/s, the axial cracks only appear on the edge of the worn surface where the delamination of the tribo-layers occurs. At the middle of the worn surface, where the surface is smooth and there is severe wear, no axial cracks appear.

### 3.4. Cross-Sectional Morphology of Seal Fin

**Figure 7:**
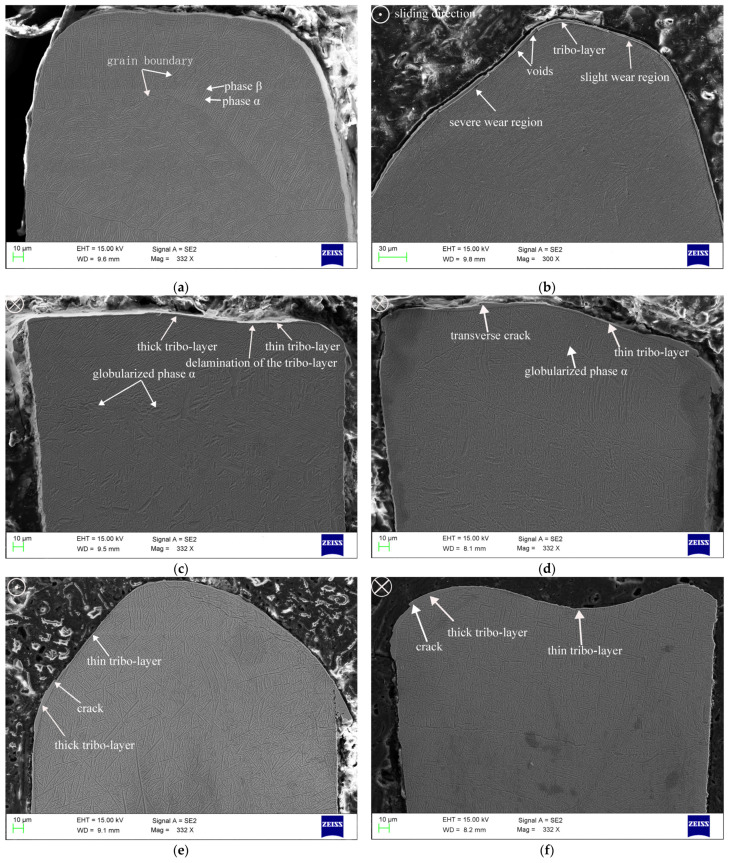

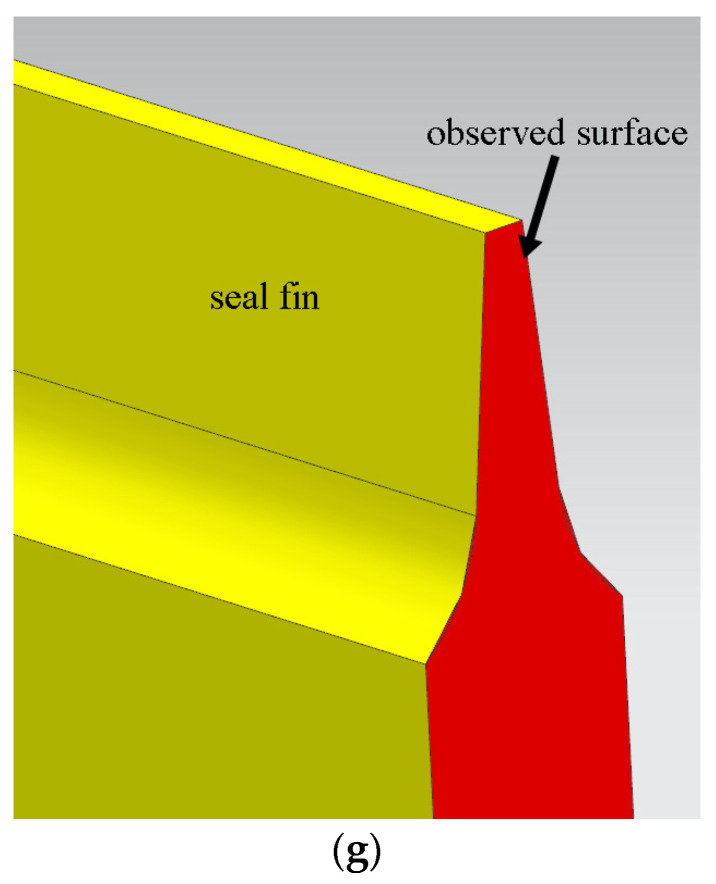
Cross-sectional morphology of seal fin at different incursion rates. (**a**) The original seal fin; (**b**) 20 µm/s; (**c**) 60 µm/s; (**d**) 100 µm/s; (**e**) 140 µm/s; (**f**) 180 µm/s; (**g**) observed surface.

The seal fin was cut perpendicular to the sliding direction to observe the cross section (red surface in Figure 7g). The cross-sectional morphology of the seal fin at different incursion rates is shown in Figure 7. The white circle in the upper-left corner in Figure 7 presents the sliding direction. The tribo-layer on the contact surface is the most obvious feature and appears at all incursion rates. However, the tribo-layer at different incursion rates has different features. 

The original seal fin shown in Figure 7a does not have tribo-layer on the top, and the substrate microstructure shows a basket-weave microstructure. At 20 μm/s, the wear of the seal fin was uneven. The height of the left part of the seal fin in Figure 7b is lower than that of the right part of the seal fin, which indicates that the wear of the left part of the seal fin is severe. The left part of the seal fin shown in Figure 7b corresponds to region A in Figure 6b, and the right part corresponds to region B in Figure 6b. Hence, region A and region B in Figure 6b are regions of severe wear and slight wear, respectively. The tribo-layer with a thickness of about 3.5 μm covers both the severe wear region and the slight wear region. However, the tribo-layer does not firmly adhere to the substrate demonstrated by the voids shown in Figure 7b. The substrate structure is the same as the original microstructure. Compared with the morphology shown in Figure 6b, the axial cracks are more obvious on the severe wear region (region A in Figure 6b). At 60 μm/s, the tribo-layer is thinner than that at 20 μm/s. The delamination wear is obvious, resulting in the different thickness of the tribo-layer shown in Figure 7c. The globularization of phase α is very distinct on the substrate, which indicates that frictional heat affects the substrate. At 100 μm/s, the tribo-layer firmly adheres to the substrate. The thickness of the tribo-layer is uneven. Transverse cracks appear on the thick tribo-layer, as shown in Figure 7d, which indicates that delamination occurs. Compared to the morphology shown in Figure 6d, it can be confirmed that the axial cracks appear on the thick tribo-layer where the delamination wear is severe. The substrate microstructure also presents the globularization of phase α. However, the degree of phase α globularization is slighter than that at 60 μm/s. At 140 μm/s, the tribo-layer covers the top of the seal fin. The tribo-layer firmly adheres to the substrate and the transverse cracks appear on the thick tribo-layer. The substrate microstructure has little difference from the original microstructure, which indicates that the frictional heat does not have significant influence on the substrate. At 180 μm/s, the wear of the middle of the seal fin is severe. Due to the rapid wear, the tribo-layer on the middle of the seal fin is very thin, with a thickness of only 0.7 μm. At the side of the seal fin where the wear is relatively slight, the tribo-layer with transverse cracks is thick. Delamination wear occurs and axial cracks appear at the side of the seal fin compared with the morphology shown in Figure 6f. The substrate microstructure is also the same as the original microstructure, which indicates that frictional heat does not affect the substrate.

### 3.5. EDS Analysis

**Figure 8:**
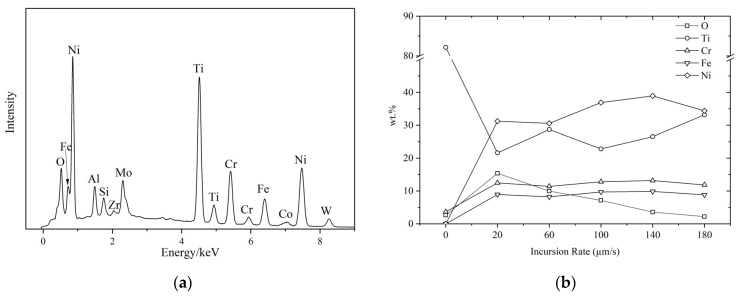
(**a**) The EDS spectrum at 100 μm/s; (**b**) the weight percentage of five elements at different incursion rates.

EDS analysis of the worn surface of seal fin rubbing under various incursion rates is illustrated in Figure 8. The EDS spectra at different incursion rates are similar. Hence, only the EDS spectrum at 100 μm/s is shown in Figure 8a. The EDS spectra show that the worn surface of the seal fin mainly contains O, Ti, Cr, Fe, and Ni elements. The weight percentages (wt.%) of these five elements at different incursion rates are shown in Figure 8b. The incursion rate of 0 µm/s shown in Figure 8b represents the original seal fin. The Cr, Ni and Fe increase significantly compared with the original seal fin, especially for Ni. Cr, Ni and Fe are mainly contained in the honeycomb, which indicates that some materials were transformed to the seal fin from the honeycomb during the rub. The wt.% of Cr and Fe do not have much difference at different incursion rates. The wt.% of Ni reaches the maximum at 140 μm/s, and then slightly decrease at 180 μm/s. The wt.% of O is maximum at 20 μm/s because the rubbing time was longest, and the tribo-layer had enough time to be oxidized. The wt.% of O then decreases with the increasing incursion rate. One reason for the decrease in the wt.% of O is that the rubbing time was shorter, and the tribo-layer did not have enough time to be oxidized. The other reason is that the tribo-layer was rapidly worn off at high incursion rates, as shown in Figure 5. Ti exists on the worn surface. However, the wt.% of Ti is much lower than that on the original seal fin surface.

**Figure 9:**
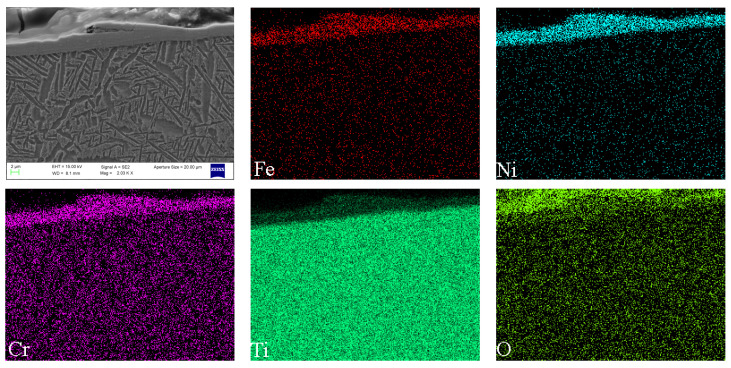
The EDS mappings of the typical cross-sectional surface of a seal fin (100 μm/s).

The EDS mappings of the typical cross-section surface of seal fin are shown in Figure 9. It is clearly shown that the concentration of Cr, Ni, and Fe can be observed in the tribo-layer. The tribo-layer also contained Ti and O. However, the wt.% of Ti is much lower than that in the substrate, and the wt.% of O is also very low. The results of the EDS mappings indicate that the compositions of the tribo-layer contain the elements from both the seal fin and the honeycomb, and the oxidation degree of the tribo-layer is low.

## 4. Discussion

### 4.1. The Wear Mechanism at Different Incursion Rates

High rubbing speed is a typical feature of the rub between seal fins and honeycomb. Large amounts of frictional heat will be generated during the rub at high incursion rates, resulting in the rapid increase in temperature as shown in Figure 4. Straffelini and Molinari reported that frictional heat effects become of primary importance for the formation of tribo-layers on the surface when sliding speed is high [15]. Abdel-Aal [16] presented an alternative theory for the thermal events that take place during the sliding of metals. He pointed out that the anisotropic of thermal conduction causes thermal energy accumulation within the contact layer, which is correlated to the transition of the wear mechanism. He used this theory to analysis the wear data in ref 15. The results showed that the flash temperature increases with the sliding speed, resulting in the decrease in normal thermal conductivity and normal heat dissipation capacity. As a result, the frictional heat will be more prone to accumulate in the lateral plane (plane of sliding) rather than conduct to the bulk material. If the amount of heat accumulated is less than the critical amount needed to instigate delamination, the oxidation wear regime will be dominant, or delamination will operate. In his further study [17], he pointed out that the frictional heat conducted to the contact layer can dissipate in three parts. Part of this frictional heat will be conducted towards the bulk of the material Q_cond_, another part will be consumed in raising the internal energy within the contact layer Q_st_, and part will be transferred away by means of the wear particles (mass leaving the system). When the heat dissipation capacity of the contact layer, which is defined as the sum of Q_cond_ and Q_st_; if this lower than the input frictional heat, the wear will be more severe. In the tests shown in this paper, the rubbing speed remained constant and the incursion rate changed. The high incursion rate means that the incursion depth per revolution is deeper. As a result, more frictional heat will be generated at the same time; it was proved in Figure 4 that the time needed to reach a high temperature is shorter with the incursion rate. It can be deduced that large amounts of frictional heat generated in a short time will tend to accumulate in the contact layer rather than conduct to the bulk of the seal fin with the incursion rate, resulting in a high surface temperature and high temperature gradient for a Ti17 seal fin. The yield strength of the Ti17 alloy is very sensitive to temperature and the yield strength will decrease greatly at high temperature [18]. Hence, the softened contact layer at high temperature will also facilitate delamination, causing that the wear rate to increase with the incursion rate, proved by the wear rate shown in Figure 5.

Based on the above theory and the scanning electron microscopy results, the wear mechanisms at different incursion rates can be qualitatively analyzed. At 20 μm/s, the long rubbing time and the less frictional heat generation cause the temperature to increase slowly. The heat dissipation capacity of the contact layer is higher than the input frictional heat. The tribo-layer readily forms on the surface and remains intact during the rub. The tribo-layer is oxidized due to the long rubbing time and low wear rate—it was proved in Figure 8b that the wt.% of O is highest at 20 μm/s. The tribo-layer protects the titanium alloy from metal wear, resulting in the low wear rate which has already been proven [19,20]. On the one hand, formation of the tribo-layer consumes the generated frictional heat; on the other hand, it prevents the frictional heat from conducting to the bulk of the seal fin. As a result, the substrate temperature does not increase greatly, and the substrate microstructure does not change. Pauschitz [21] pointed out that the tribo-layer can be classified into three kinds: (1) a transfer layer with low oxygen and the same composition as the counterface material; (2) a mechanically mixing layer (MML) with low oxygen, and the compositions situated between the pin and counterface at higher temperature; (3) a composite layer with high oxygen, hard and brittle, formed at higher temperature. According to the EDS analysis results, the tribo-layer on the worn surface had a low wt.% of O, and compositions from both the honeycomb and the seal fin. Apparently, the tribo-layer is a mechanically mixing layer (MML). To sum up, oxidation wear and adhesive wear are the dominant wear mechanisms at 20 μm/s. At 60 μm/s, the intensity of the frictional heat generation increases, leading to the accumulation of the frictional heat on the contact layer. At the beginning, the accumulated frictional heat is lower than the heat dissipation capacity so that the tribo-layer forms and thickens. When the accumulated frictional heat exceeds the heat dissipation capacity, the delamination of the tribo-layer occurs. Once the delamination wear occurs, the generated wear particles will carry away the heat and hence reducing the heat accumulated in the contact layer. The residual heat in the contact layer becomes lower than the critical amount needed to instigate delamination, and the delamination wear is terminated. Then, as the rubbing progresses, the above process is repeated. Hence, the delamination occurred periodically, proved by the periodical large peaks in the curves of rubbing force and rubbing temperature shown in Figure 4b. The periodical delamination indicates that the frictional heat generation and the heat dissipation were in equilibrium at 60 μm/s. At the same time, a large amount of the frictional heat conducted to the bulk of the seal fin, leading to the temperature increasing, which induced the globularization of phase α on the substrate, as shown in Figure 7c. The investigation in [22] pointed out that the thermal cycling below the β-transus will induce the globularization of the α phase, while the isothermal heat only induces the small fraction of globularization of the α phase. The microstructure obtained in [22] under thermal cycling is very similar to that in Figure 7c. This proves the fluctuation of the rubbing temperature shown in Figure 4b is the reason for the globularization of phase α on the substrate. Hence, delamination wear and phase α globularization on the substrate are dominant at 60 μm/s. At 100 μm/s, more frictional heat is generated, and the frictional heat that exceeds the heat dissipation capacity of the contact layer is more than that at 60 μm/s. As a result, the frictional heat accumulated in the contact layer is more than that at 60 μm/s, meaning that the delamination wear is more likely to occur. This is proved by the rapped tracks shown in Figure 6d and cracks on the tribo-layer shown in Figure 7d. Due to the rapid wear of the seal fin that carries away heat from the contact layer, less frictional heat conducts to the bulk of the seal fin than that at 60 μm/s, causing the temperature at the substrate to be lower than that at 60 μm/s. Additionally, the fluctuation of the rubbing temperature is less than that at 60 μm/s. As a result, the phase α globularization occurs on the substrate, but the degree of the phase α globularization is lower than that at 60 μm/s. On the whole, the delamination wear is still dominant at 100 μm/s, accompanied by substrate phase α globularization. At 140 μm/s and 180 μm/s, the intensity of frictional heat is too high, causing the high temperature on the contact layer and high temperature gradient on the substrate. The large amount of frictional heat accumulates in the contact layer and softens the contact layer. The strength of the softened material is low, leading to rapid wear of the seal fin under high load. Due to the rapid wear which carries away large amounts of heat, the frictional heat conducted to the bulk of the seal fin is small. As a result, the temperature of the substrate is lower than that at 60 μm/s and 100 μm/s. The fluctuation of the rubbing temperature is also not obvious. Hence, the substrate microstructures of the seal fin at 140 μm/s and 180 μm/s, shown in Figure 7e,f, respectively, have little difference compared to that of original seal fin—especially at 180 μm/s. The softened tribo-layer and the substrate are firmly adhered to each other and embedded into each other shown in Figure 7e,f. The tribo-layer is thin at the severe wear region and cannot protect the seal fin. As a result, the metal wear may occur. Relatively, the worn morphology at 140 μm/s shown in Figure 6e presents more delamination wear features (rough surface), while the worn morphology at 180 μm/s shown in Figure 6f presents more metal wear features (smooth surface).

### 4.2. The Causes of the Axial Cracks

Combining the worn morphology shown in Figure 6 and the cross-sectional morphology shown in Figure 7, it can be confirmed that the axial cracks on the worn surface are highly related to the tribo-layer. When the tribo-layer is thick and has transverse cracks, the axial cracks on the worn surface are obvious. This is more pronounced at 100 μm/s and 180 μm/s, where the axial cracks disappear at the thin tribo-layer. At 20 μm/s, the tribo-layer covers the whole surface, and the thickness is even. However, the axial cracks are obvious at severe wear region A, where the tribo-layer is subjected to high rubbing force and temperature. This means that axial cracks are caused by the tribo-layer cracking under periodic thermomechanical stress. The rubbing time also affects the cracking of the tribo-layer. Due to the constant rubbing speed and incursion depth, the low incursion rate means the long rubbing time and thus the tribo-layer will be subjected to more periodic thermomechanical stress, which will accelerate the axial crack propagation. Hence, the axial cracks at 20 μm/s are wide and deep, while the axial cracks at the side of the seal fin at 180 μm/s where the thickness of the tribo-layer is similar to that at 20 μm/s are narrow and shallow, as shown in Figure 6b,f. Another explanation for the absence of the axial cracks at high incursion rate is the competition and restriction coupling mechanism between wear and cracks [23,24]. Rapid wear of the tribo-layer will suppress axial crack generation and propagation, proved by the middle region of the seal fin at 180 μm/s and shown in Figure 6f where the wear is severe.

On the whole, the thick tribo-layer generated by oxidation wear and adhesive wear, such as at 20 μm/s, is more prone to crack under periodic thermomechanical stress while the delamination wear and metal wear inducing the severe wear will suppress the generation and propagation of axial cracks at high incursion rate. It is to be noted that at 20 μm/s, the tribo-layer does not firmly adhere to the substrate. The propagation of axial cracks may only induce the peeling of the tribo-layer. However, at high incursion rate, such as 180 μm/s, the tribo-layer and the substrate are embedded into each other as shown in Figure 7f. The propagation of axial cracks may induce the cracking of the substrate which will endanger the security of the aero-engine. The tribo-layer on the seal fin is very thin (maximum thickness of tribo-layer at 20 μm/s is about 3.5 μm); therefore, polishing the surface of the seal fin to remove the tribo-layer after the rub will be the practical method to remove the axial cracks.

### 4.3. The Relationship between the Wear Mechanism and the Rubbing Force and Temperature

With the increasing incursion rate, the wear mechanism of the Ti17 seal fin changes from adhesive wear and oxidation wear to delamination wear and then to metal wear. The change of the wear mechanism has a profound effect on the rubbing force and temperature. At 20 μm/s, the tribo-layer is thick and intact. Adhesive wear and oxidation wear are dominant. As a result, the curves of rubbing force and temperature are smooth with the rubbing time. The fluctuation on the curves at the end of the rub may be due to the slight delamination occurring at the region between the severe wear region and the slight wear region, as shown in Figure 6b. At 60 μm/s, the delamination wear is the dominant wear mechanism. The delamination region on the tribo-layer, which is lower than the undelamination region, reduces the contact area and creates a small gap between seal fins and the honeycomb, resulting in the decrease in rubbing force and temperature. As a result, accompanied by the periodical delamination of the tribo-layer, the periodical fluctuation on the curves of rubbing force and the temperature appears. At 100 μm/s, the delamination wear is still the dominant wear mechanism. However, the high incursion rate will quickly compensate for the small gap between contact pairs due to delamination wear. This is the reason why the duration of the troughs on the rubbing force and temperature curves is shorter than that at 60 μm/s. At 140 μm/s, the delamination wear is still dominant; however, the delamination region is very rough compared to those at 60 μm/s and 100 μm/s. The rough delamination region shown in Figure 6e indicates that the delamination of the tribo-layer mainly occurs in a small region, which is different from the large-scale delamination region at 60 μm/s and 100 μm/s. As a result, small peaks appear on the curves of rubbing force and temperature instead of large peaks. At 180 μm/s, the metal wear is dominant and the worn surface is very smooth, with a thin tribo-layer at the middle of the seal fin. The delamination wear only appears at the side of the seal fin. Hence, the rub between the seal fin and the honeycomb is continuous, resulting in less fluctuation and no large peaks on the curves of the rubbing force and temperature. 

From the above discussion, the delamination wear will induce the large fluctuation of the rubbing force and temperature, while the metal wear will smoothen the curves of the rubbing force and temperature. In aero-engines, it is hard to check the seal fin in time, but it is relatively easy to acquire force data or temperature data during the aero-engine operation. The results discussed in this section provide a method to judge the wear mechanism of the seal fin based on the data of force or temperature. Combined with the relationship between the wear mechanism and the axial cracks, the condition of the tribo-layer and the form of the axial cracks on the seal fin after the rub can also be inferred.

## 5. Conclusions

The rub tests between the labyrinth seal fin and the honeycomb at different incursion rates were performed under rubbing speed of 380 m/s and temperature of 350 °C. The wear mechanism of the seal fin and the seal fin damage was analyzed and discussed based on the results of wear rate, worn morphology, EDS results, rubbing force and temperature. The following conclusions can be drawn from the present study.

1. The wear mechanism of the seal fin changes from oxidation wear and adhesive wear at low incursion rates to delamination wear and metal wear at high incursion rates. When oxidation wear and adhesive wear are dominant, the tribo-layer on the contact surface is thick and intact. The tribo-layer protects the seal fin from severe wear. When the delamination wear and metal wear are dominant at high incursion rates, the thin tribo-layer cannot protect the seal fin, resulting in the high wear rate. As a result, the wear rate of the seal fin increases, and the wear rate shows an exponential growth trend with the increasing incursion rate.
2. The globularization of α phase occurs on the substrate at 60 μm/s and 100 μm/s, which indicates that frictional heat has a great influence on the substrate. Relatively, the degree of α phase globularization at 60 μm/s is higher than that at 100 μm/s. At high incursion rates, such as 140 μm/s and 180 μm/s, the substrate microstructure is the same as the original microstructure because the rapid wear will carry away the frictional heat.
3. The axial cracks appear on the worn surface due to the cracking of the tribo-layer under periodic thermomechanical stress during the rub. The crack propagation is inhibited at 180 μm/s due to the rapid wear of the tribo-layer and short rubbing time. However, at high incursion rates, the tribo-layer and the substrate adhere to and are embedded into each other. The propagation of the axial cracks may lead to the cracking of the substrate, which will endanger the security of the aero-engine. Polishing the worn surface of the seal fin after the rub to remove the cracked tribo-layer is a practical method to eliminate the axial cracks.
4. The changes of radial force and temperature with incursion depth correspond to the wear mechanism of seal fins. The periodical large peaks on the curves of rubbing force and temperature indicates that the delamination wear is dominant, while the relatively smooth curves indicate that the metal wear or oxidation wear is dominant.

## Figures and Tables

**Table 1:** The composition of Ti17 alloy (wt.%).

Composition	Ti	Al	Cr	Zr	Mo	Sn
Content (%)	balance	5.03	3.88	1.99	4.02	2.07

**Table 2:** The composition of Hastelloy X alloy (wt.%).

Composition	Ni	Cr	Fe	Mo	Co	Al	W	Si	C	P	Cu
Content (%)	balance	21.74	19.18	8.46	1.31	0.13	0.65	0.24	0.066	0.014	0.08

**Table 3:** Test parameters.

Test No.	Incursion Speed (V_inc_, μm/s)	Temperature (T, °C)	Rubbing Speed (V_t_, m/s)	Incursion Depth (D_p_, μm)
I-1	20	350	380	1500
I-2	60			
I-3	100			
I-4	140			
I-5	180			

## Data Availability

Data sharing not applicable.
